# scDC: single cell differential composition analysis

**DOI:** 10.1186/s12859-019-3211-9

**Published:** 2019-12-24

**Authors:** Yue Cao, Yingxin Lin, John T. Ormerod, Pengyi Yang, Jean Y.H. Yang, Kitty K. Lo

**Affiliations:** 1 School of Mathematics and Statistics, The University of Sydney, Sydney, NSW 2006 Australia; 2 Charles Perkins Centre, The University of Sydney, Sydney, NSW 2006 Australia; 3 Children’s Medical Research Institute, Faculty of Medicine and Health, The University of Sydney, Sydney, NSW 2145 Australia

**Keywords:** Single cell, RNA-seq, scRNA-seq, Composition analysis

## Abstract

**Background:**

Differences in cell-type composition across subjects and conditions often carry biological significance. Recent advancements in single cell sequencing technologies enable cell-types to be identified at the single cell level, and as a result, cell-type composition of tissues can now be studied in exquisite detail. However, a number of challenges remain with cell-type composition analysis – none of the existing methods can identify cell-type perfectly and variability related to cell sampling exists in any single cell experiment. This necessitates the development of method for estimating uncertainty in cell-type composition.

**Results:**

We developed a novel single cell differential composition (scDC) analysis method that performs differential cell-type composition analysis via bootstrap resampling. scDC captures the uncertainty associated with cell-type proportions of each subject via bias-corrected and accelerated bootstrap confidence intervals. We assessed the performance of our method using a number of simulated datasets and synthetic datasets curated from publicly available single cell datasets. In simulated datasets, scDC correctly recovered the true cell-type proportions. In synthetic datasets, the cell-type compositions returned by scDC were highly concordant with reference cell-type compositions from the original data. Since the majority of datasets tested in this study have only 2 to 5 subjects per condition, the addition of confidence intervals enabled better comparisons of compositional differences between subjects and across conditions.

**Conclusions:**

scDC is a novel statistical method for performing differential cell-type composition analysis for scRNA-seq data. It uses bootstrap resampling to estimate the standard errors associated with cell-type proportion estimates and performs significance testing through GLM and GLMM models. We have made this method available to the scientific community as part of the *scdney* package (**S**ingle **C**ell **D**ata I**n**t**e**grative Anal**y**sis) R package, available from https://github.com/SydneyBioX/scdney.

## Background

Tissues are composed of many heterogeneous cell-types and differences in cell-type proportions often carry biological significance. For example, cell-type compositional differences can shed light on disease mechanisms [1], immune response in cancer [2] and developmental processes [3]. In recent years, single cell RNA sequencing (scRNA-seq) has become an increasingly popular technology as it allows the cell-type identity of each individual cell sequenced to be inferred. This has enabled generation of insights through comparison of cell-type composition between samples.

There are many challenges associated with estimating cell-type composition. First, there could be technical biases for each cell-type. Certain cell-types may be more prone to damage under existing scRNA-seq protocols [4], leading to bias and perhaps a larger variance in cell-type proportions. Second, there are many proposed methods for cell-type identification and none of them guarantees perfect identification accuracy. A systematic evaluation of 14 common clustering algorithms revealed differences in performance and clustering result [5, 6]. A further evaluation on similarity metrics in clustering algorithms illustrates further inconsistency in results due to the different metrics used for measuring differences in cell transcriptomes [7]. In addition, as the compositions sum to one, this transforms the data from the Euclidean space into a simplex space. Canonical statistical tests such as t-test may not be reliable, as these are not designed for relative proportion data [8]. These issues all highlight the difficulty and uncertainty associated with cell-type identification. However, there is a current lack of methodological research on cell-type compositional analysis for single cell data. Whilst some research papers draw observations on compositional differences of cell-types between conditions [1, 3, 9], such observations are not accompanied by a measure of uncertainty associated with the estimates. In contrast to the lack of such studies in the field of single cell analysis, compositional analysis has been an active and ongoing area in microbiome research, due to the compositional nature of microbiome data. Dirichlet – multinomial models have been used extensively for microbiome research, in which Dirichlet Monte Carlo procedures are used to obtain posterior distributions of sample proportions [10–13]. However, due to the small sample size of scRNA-seq data, fitting a Dirichlet multinomial is not currently feasible.

In this paper, we present **s**ingle **c**ell **D**ifferential **C**omposition (scDC), a method for estimating uncertainty in cell-type proportions via bootstrapping. Our method provides a bias-corrected estimate of cell-type proportions confidence intervals, and by performing clustering within each bootstrap iteration it also captures the uncertainty associated with cell-type identification. In scRNA-seq experiments with small sample sizes, such estimates of cell-type proportion uncertainties would be useful in determining the significance of cell-type compositional differences between conditions. In the future where multi-sample comparative scRNA-seq experiments are likely to become common place, our method offers a statistically robust way to propagate uncertainty in downstream analysis. We demonstrate this using two synthetic datasets derived from published experimental data. Finally, we implemented scDC as part of the *scdney* R package which is freely available from https://github.com/SydneyBioX/scdney.

## Results

### Overview of single cell differential composition analysis (scDC)

**Fig. 1:**
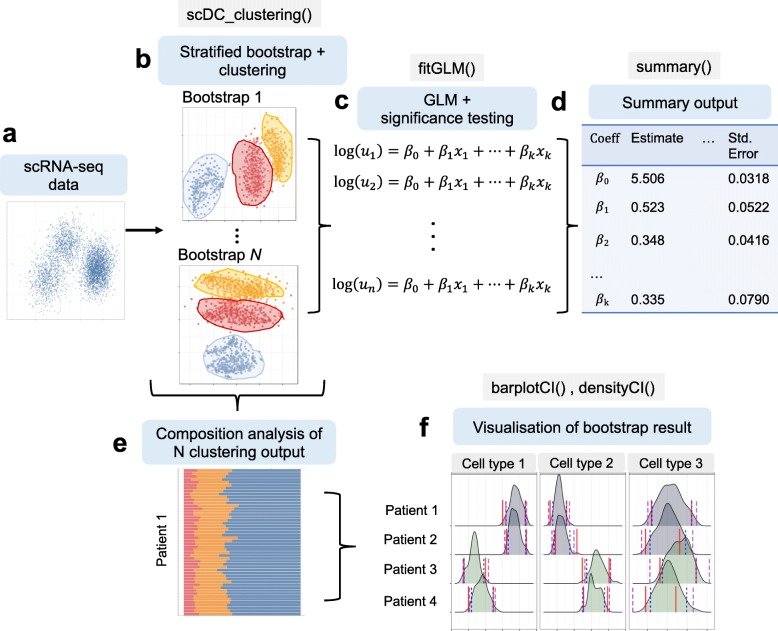
Overview of scDC workflow. This illustrates the main components of the scDC procedure. The key functions have been included on top of each sub-figure, where relevant. **a** Single cell data is collected and analysed. Publicly available experimental data is obtained. Simulated data is generated using R package *PowSimR*. **b** This corresponds to step 1 and 2 of the 4-step procedure. Resampled data is generated using stratified bootstrap and then clustered using clustering algorithm. Each cluster is matched to cell-type using reference cell labels in the original dataset. This step is repeated *n* times. **c** In step 3 and 4, cell count output from each bootstrap is fitted using GLM. The coefficient estimates from each individual GLM model are pooled using Rubin’s rules and tested for significance. **d** User can extract the overall estimates of statistics. **e** Each bootstrap re-sampling gives an estimated distribution of cell-types composition for each patient. **f** the result can be visualised graphically

We developed scDC (workflow shown in Fig. 1), a novel approach based on bootstrap resampling, to perform differential cell-type composition analysis. Let *Y*_*ij*_ be a matrix of cell counts from *i*=1,…,*C* cell-types and *j*=1,…,*R* subjects. The first step of scDC involves stratified bootstrap resampling where we sampled with replacement *N*_*j*_ cells from the cell count matrix *Y*_*ij*_ for subject *j*. Stratification by subject is important because it ensures unbiased bootstrap estimates and captures subject to subject variability. Let $Y^{*}_{bj}$ be the *b*^*th*^ bootstrap sample for the *j*^*th*^ subject. Subsequently, for each bootstrap iteration, we combined resampled cell counts $Y^{*}_{b1},\ldots,Y^{*}_{bR}$ for all *R* subjects.

The next step involved cell-type identification of each bootstrap sample using clustering. Here, we performed PCA dimension reduction followed by k-means clustering (using Pearson correlation as distance metric [7]). For each bootstrap sample, this generated cell-type proportions $p^{*}_{b1},\ldots p^{*}_{bC}$ which we later used to calculate bootstrap standard errors (SE) for each of the cell-type proportions *p*_1_,…,*p*_*C*_. In parallel, for experiments with two conditions A and B, to assess changes in cell-type compositions between conditions, we modelled the data using a log-linear model accounting for cell-types, conditions, subjects and interaction effects between cell-types and conditions. A log-linear model was fitted to each bootstrap sample $Y^{*}_{b}$ and the results were pooled using Rubin’s pooled estimate (see “Methods” section) [14]. This log-linear model can also be fitted as a mixed effect model by giving each subject a random effect and both options are available in our scDC framework.

### Stratified bootstrap procedure provides good estimation of sampling error for cell-type proportions

**Fig. 2:**
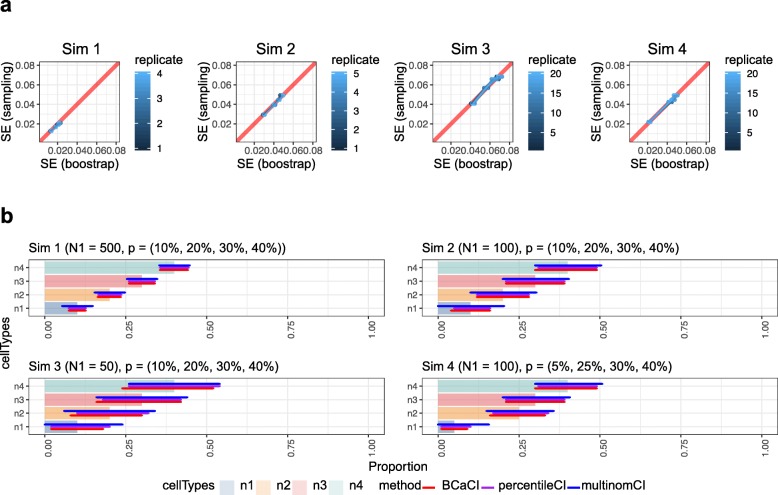
Stratified bootstrap provides good estimate of standard error. **a** Using four simulated datasets, stratified bootstrap was performed to estimate cell-type proportions in each bootstrap. Each dot on the figure represents a pair of value containing the mean standard error of bootstrap (x-axis) and standard error from sampling (y-axis). **b** Each horizontal bar shows confidence interval associated with proportion estimate

First, we examined the validity of our stratified bootstrap procedure using simulation data (Table 1). In Fig.2a each point represents the sampling standard error and the bootstrap standard error associated with a particular subject. The figure clearly shows a high concordance between bootstrap standard error and sampling standard error. Therefore, bootstrap resampling should provide a reasonable estimate of uncertainty for cell-type proportion.

**Table 1:** Simulation cases with one condition

Datasets	*C*	Number of replicates (*R*)	*p*_1_	*p*_2_	*p*_3_	*p*_4_
Sim1	500	10	0.1	0.2	0.3	0.4
Sim2	100	50	0.1	0.2	0.3	0.4
Sim3	50	100	0.1	0.2	0.3	0.4
Sim4	100	50	0.05	0.25	0.3	0.4

Four simulation datasets were made, each dataset containing 5000 cells. *C* represents the number of cells from one replicate. *p*_1_,*p*_2_,*p*_3_,*p*_4_ corresponds to the proportion of the four cell-types *Cell*_1_, *Cell*_2_, *Cell*_3_ and *Cell*_4_ respectively

### Bias-corrected and accelerated (BCa) bootstrap confidence interval for single subject cell-type proportions

We examined the effectiveness of various approaches to estimate confidence intervals (CI) associated with cell-type proportions at the single subject level. Cell-type proportions from a single cell experiment can be modeled using a multinomial distribution with *C* proportion parameters *p*_1_,…,*p*_*C*_. The simple standard error (SE) measure, commonly used to illustrate error bars, assumes symmetric CIs and therefore is not an appropriate representation of the CI of cell-type proportions. The SE estimate is especially problematic for rare cell-types, as it can generate CI estimates with endpoints that extend outside [0,1]. Existing approaches to estimate CIs for multinomial proportions require large expected cell counts [15]. For rare cell-types, cell counts may not be adequate to ensure the appropriate overall coverage probability.

To address this issue, we considered three methods to estimate CI of cell-type proportions: (1) the model-based approach of May and Johnson (2000) [16, 17], which constructs simultaneous CIs for multinomial proportions; (2) the bootstrap percentile method, and (3) the bootstrap BCa method [18]. Figure 2b shows CI estimates using these three methods on four sets of simulated data. Comparing simulation data Sim1 with 500 cells per replicate, Sim2 with 100 cells per replicate and Sim3 with 50 cells per replicate, it is clear that the width of all three types of confidence intervals increases with decreasing number of cells. The width of BCa CI is similar to multinomial and percentile in all cases, but is significantly shorter when there was presence of rare cell-types and small number of cells per replicate, as shown by the cell-type n4. This suggests that BCa provides better estimates for rare cell-types.

### scDC correctly estimates the cell-type proportions and corresponding standard error in simulation data

**Fig. 3:**
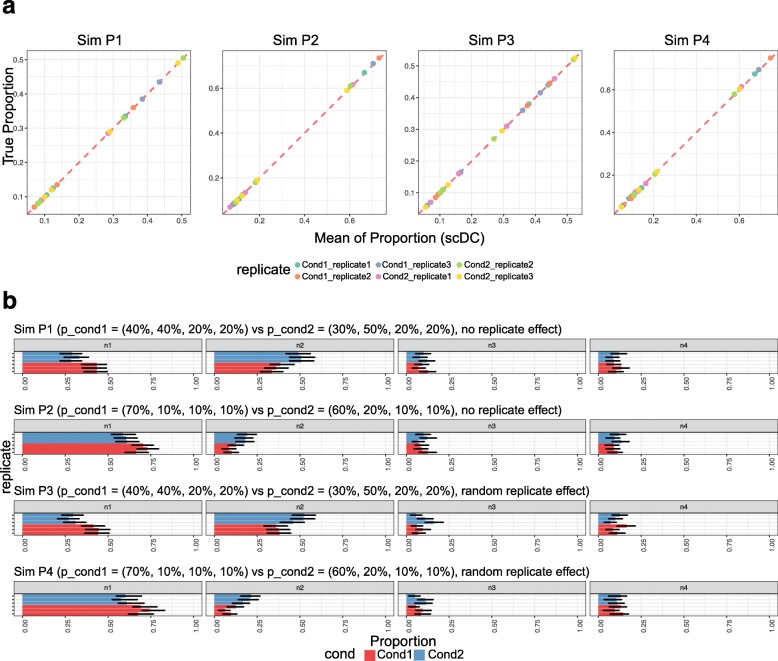
scDC on simulated dataset. scDC has been applied to four simulated datasets. **a** Each dot represents a pair of values containing the mean proportion estimate calculated from scDC (x-axis) and the true proportion from the simulation data (y-axis). Across all four datasets, all dots lie on the diagonal line, even when the proportion of a cell-type is as low as 5%. This demonstrates that scDC is highly accurate in estimating cell-type composition. **b** Each simulated dataset contains two conditions, represented suing colours red and blue. The bar chart shows proportion estimates of each cell-type *n*1 to *n*4 for each subject in the dataset. The horizontal black line represents the BCa confidence interval associated with the proportion estimate

Using a series of simulation data, we evaluated whether scDC can accurately recover true cell-type proportions and the corresponding standard errors within each condition and subject. For simulated data SimP1 and SimP2, we simulated the situation where biological replicates are available; for simulated data SimP3 and SimP4, we also modelled the subject to subject variability as random effects. The detailed simulation settings are listed in Table 2 and described in detail in “Methods” section. Figure 3a shows that in all four simulations, scDC correctly recovers the true cell-type proportions; Fig. 3b shows the corresponding bootstrap confidence intervals calculated using the BCa method. Visually, the confidence intervals overlap between conditions where cell-type proportions differ by 10%. When we used GLMs to test for significance, we found that there is significant interaction effect for cell-type 2 in simulation data SimP1 (*p*=0.01; Table 3) but not for cell-types 3 and 4. This shows concordance with the true underlying model.

**Table 2:** Simulation cases with two conditions

	Condition 1				Condition 2			
Datasets	*p*_11_	*p*_21_	*p*_31_	*p*_41_	*p*_12_	*p*_22_	*p*_32_	*p*_42_
SimP1	0.4	0.4	0.2	0.2	0.3	0.5	0.2	0.2
SimP2	0.7	0.1	0.1	0.1	0.6	0.2	0.1	0.1
SimP3	0.4	0.4	0.2	0.2	0.3	0.5	0.2	0.2
SimP4	0.7	0.1	0.1	0.1	0.6	0.2	0.1	0.1

Four simulation datasets are made, each contains 6000 cells. *p*_*ij*_ corresponds to the proportion of cell-type in cell-type *i* and condition *j*

**Table 3:** Summary of pooled estimates from fitting GLMM to bootstrap samples from simulation data *SimP1*

	Mixed effect model - Simulation data SimP1				
	Estimate	std.error	Statistic	df	*p*.value
(Intercept)	4.32	1.24	3.49	6.11	0.01
*Cell*_2_	-0.17	0.14	-1.22	5.86	0.26
*Cell*_3_	-1.50	0.23	-6.64	5.52	0.00
*Cell*_4_	-1.35	0.19	-7.04	6.79	0.00
Condition2	-0.02	1.81	-0.01	5.59	0.99
*Cell*_2_:Condition2	0.68	0.19	3.53	6.20	0.01
*Cell*_3_:Condition2	0.41	0.32	1.29	5.55	0.23
*Cell*_4_:Condition2	0.25	0.26	0.95	7.97	0.37

We also examined scDC’s performance on estimating cell-types proportions in datasets where there are highly correlated cell-types and rare cell-types, as these situations are often observed in real experimental datasets. Using eight simulated datasets SimS1 to SimS8, we demonstrated that scDC is highly accurate in recovering rare cell-types proportion as low as 1% and is only slightly affected when there is high degree of correlation between two cell-types (Additional file 1: Figure S1). The detailed simulation settings are listed in Additional file 1: Table S2)

Lastly, we simulated a dataset composed of 4000 cells and 10 cell-types with 3 rare cell-types, replicating the size and the complexity of real experimental dataset. scDC result shown in Additional file 1: Figure S2 clearly revealed that scDC accurately recovered true cell-types proportion. In addition, we observed that BCa produced the tightest CI interval around the density distribution of proportion estimates for the three rare cell-types, n1, n2 and n3. In contrast, both percentile and multinomial CI produced much wider CI in some cases. For the other seven cell-types, the three CI estimates were similar. This is consistent with our findings in the previous subsection.

### Impact of specifying incorrect number of cell-types

**Fig. 4:**
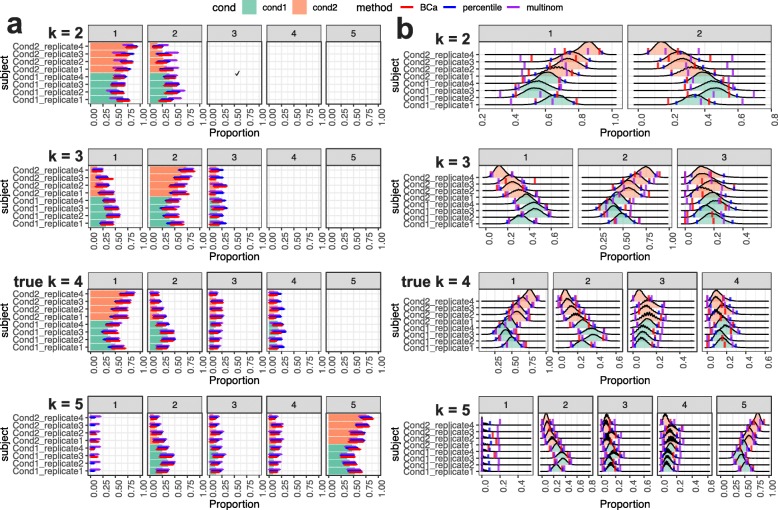
Impact of incorrectly specified numbers of cell-types. We simulated a dataset containing 4 cell-types and ran scDC with number of cell-types specified to be 2, 3, 4 and 5. **a** shows the width of the three types of confidence intervals. **b** shows the density distribution of proportion estimates

In practice, the true number of cell-types are often unknown. To investigate the impact on scDC result when the number of cell-types is incorrectly determined, we simulated a dataset containing 500 cells and 4 cell-types and evaluated scDC with the number of cell-types specified as 2, 3, 4 and 5. Figure 4 clearly shows that in all cases when the input number of cell-types is incorrect, the resulting confidence intervals are very wide. Some of the confidence intervals appeared to be out of place compared to the density distribution. Only when scDC was ran with number of cell-types being 4 did the confidence intervals form reasonably CI estimate as seen from close bands surrounding the density distributions.

### Application of scDC reveals differential cell-type proportions in two synthetic datasets

To demonstrate the applicability of our method on data containing realistic variability from single cell experiments, we examined two synthetic datasets constructed from two sets of real experimental data — Pancreas [1] and Neuronal [3] (see “Methods” section).

**Fig. 5:**
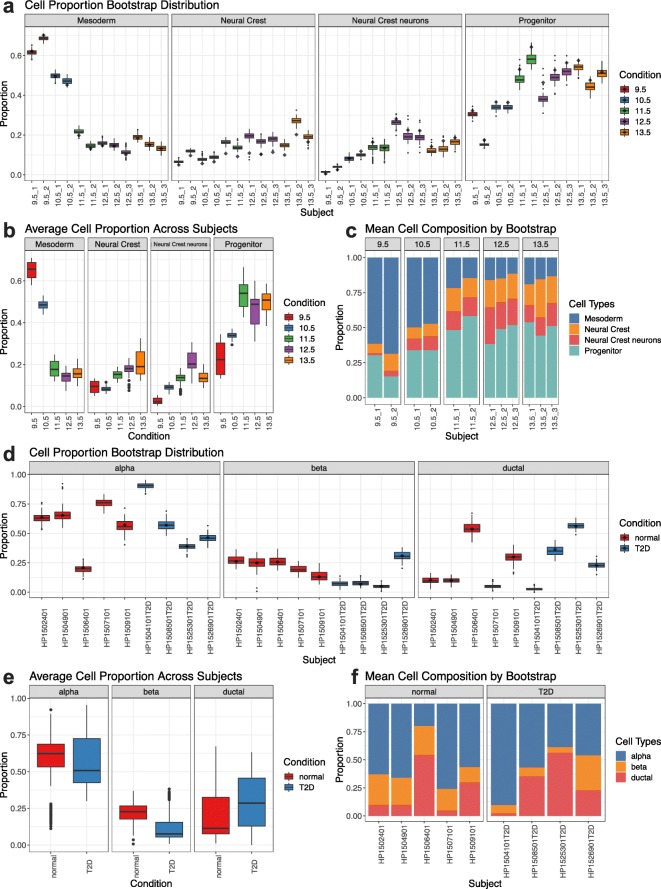
scDC on synthetic dataset. scDC has been applied on two different synthetic datasets, **a-c** shows result on neuronal dataset and **d-f** shows result on pancreas dataset. **a** Subjects are samples taken at different developmental time point. Each time point has two or three samples, as indicated by the labels on the x-axis. Boxplot represents 100 cell proportion values obtained from performing scDC. The diamond symbol in each boxplot represents the reference cell proportion value calculated from the original dataset. **b** Each boxplot is drawn by taking the mean of subjects at each time point. Thus wider boxplot indicates greater subject to subject variability. **c** This plots the mean cell composition of each subject from the 100 cell proportion values obtained from scDC and compares across the developmental time point. A trend in proportional change of the four cell-types is visible. **d** Subjects are samples taken from normal subjects and type 2 diabetes subjects. **e** Averaging across subjects for each cell-type reveals a clear difference in beta cell proportions between normal and type two diabetes subjects. **f** Compared to mouse data (**c**), human data exhibit much greater between subject variability

In the Pancreas dataset that examines subjects with type two diabetes (T2D) and healthy subjects, we observed subject to subject variation. Despite the overall proportion of beta cells being lower with subjects with T2D compared with healthy controls, our analysis confirms the original finding that 1 of the 4 subjects has a higher beta cell value. The boxplot for this patient has a small interquartile range, suggesting that there is high confidence that the high value observed with this subject is not due to an error or random chance, for example, in the clustering procedure. The overall boxplot (Fig. 5d-f) summarising the average cell proportion across subjects further shows that the interquartile range of the beta cell proportion of normal subjects is not overlapping with the interquartile range of the beta cell proportion of T2D subjects. This confirms that, despite one of the T2D subjects being an outlier, the overall beta cell proportion of T2D subjects is lower than for normal subjects. Inspecting output from random effect and fixed effect model revealed a difference in subject variability and the pooled fixed effect model also suggests the beta cell-type and subject interaction effect is significant (*p*=0.02). This again highlights that in the beta cell, there is a lower proportion in T2D compared to healthy and such difference in proportion isn’t in the alpha cell-type. The details of the GLM and GLMM results are presented in Table 4.

**Table 4:** Summary of pooled estimates from fitting GLM and GLMM to bootstrap samples from Pancreas dataset

	Fixed effect model - Pancreas				
	Estimate	std.error	Statistic	df	*p*.value
(Intercept)	4.68	0.09	52.09	9.84	0.00
Beta	-0.94	0.16	-5.87	4.10	0.00
Ductal	-1.14	0.14	-7.91	5.87	0.00
T2D	0.06	0.12	0.51	10.22	0.62
patientHP1504101_T2D	-0.28	0.14	-2.05	7.41	0.06
patientHP1504901	-0.27	0.11	-2.38	12.35	0.03
patientHP1506401	-0.39	0.12	-3.34	12.35	0.01
patientHP1507101	-0.03	0.11	-0.32	12.35	0.76
patientHP1508501_T2D	0.01	0.10	0.10	12.35	0.92
patientHP1509101	-0.87	0.14	-6.42	12.35	0.00
patientHP1525301_T2D	0.07	0.10	0.77	12.35	0.45
beta:T2D	-0.52	0.22	-2.40	5.41	0.03
ductal:T2D	0.59	0.19	3.12	5.54	0.01
	Mixed effect model - Pancreas				
	Estimate	std.error	Statistic	df	*p*.value
(Intercept)	4.37	0.12	36.20	16.21	0.00
Beta	-0.94	0.16	-5.87	5.99	0.00
Ductal	-1.14	0.14	-7.91	8.59	0.00
T2D	0.31	0.17	1.80	16.90	0.09
beta:T2D	-0.52	0.22	-2.40	7.92	0.03
ductal:T2D	0.59	0.19	3.12	8.14	0.01

Beta, ductal are the cell-type effect. T2D is abbreviation of type two diabetes effect. Subject ID followed by underscore of T2D represents type two diabetes subject. Subject ID without underscore of T2D represents normal subject

The Neuronal dataset contains samples from inbred mice at different developmental time points. With the neuronal dataset, we examined the mesoderm, neural crest, neural crest neurons, and progenitor cell-types. Here, the subject to subject variability are substantially smaller (Fig. 5a-c). Application of scDC reveals a clear trend in the relative proportion of these four cell-types. In particular, there is a clear increase in the relative proportion of progenitor, neural crest and neural crest neurons, and a decrease in the relative proportion of mesoderm. The GLM and GLMM analysis show a significant interaction effect between cell-types and time points (*p*<0.001). The details of the GLM and GLMM results are presented in Additional file 1: Table S1. The original paper examined the change in progenitor compared to neurons and stated that the percentage of progenitor increased as the percentage of neurons decreased. Our findings suggest that, by excluding neurons from analysis, there is a visible increase in the percentage of progenitor cells over time. This finding is worth further investigation, as progenitor cells is a key player in neurogenesis which gives rise to neuronal cells.

## Discussion

We present a novel statistical framework, scDC, to assess cell-type composition differences between conditions of interest. This involves developing approaches to estimate standard error of cell-type proportions estimates in scRNA-seq data via bootstrapping and adapting a missing values framework to facilitate Wald testing from bootstrap log-linear model and generalized linear mixed model analyses. By applying scDC to simulated data, we demonstrate that our method can accurately recover the correct confidence interval of cell-type proportions in an unbiased manner. Furthermore, we show in two synthetic datasets that cell-type composition differences can be accurately determined following the scDC procedure.

All confidence interval estimation procedures discussed in this paper can be extended to directly calculate the confidence interval associated with the difference in *i*^*th*^ cell-type proportions. This provides an alternative approach to the CI constructed based on *SE*_*pooled*_ that will be symmetric by default. These various CI estimation procedures generate different CIs with different coverage probabilities and interval lengths. The ability to calculate the correct coverage probability with the smallest interval length is an important component of power calculations as it leads to a reduction of sample size needed to achieve detection of differences between two cell-types.

Comparisons between the two datasets confirm the widely accepted phenomena that human data contain much higher subject to subject variability than mouse data. The lower mouse to mouse variability could be due to two factors. First, cell-types in mouse are easier to distinguish, resulting in greater stability in clustering process. Second, inbred mice might exhibit much less variability than humans. Our scDC method provides and encourages the visualization of SE associated with cell-type proportions estimates at different level. For example, in the Pancreas dataset, despite there being only one T2D subject with a higher beta cell proportion, there is still a significant interaction effect between T2D and beta cell proportions when taking account of all subject. This opens up a potential problem of hierarchical compositional analysis where one identifies the proportion of population within T2D that demonstrates a higher beta-cell proportion versus the proportion of population within T2D that has a lower beta-cell proportion, and what sample size of subject is required to achieve this.

As with all bootstrap approximations, we are limited by the initial sampling procedure. This is more complicated in data with samples from different human subjects, where the cell-type proportions in each sample vary greatly. In addition, comparing Sim1 and Sim3 from Fig.2b, it is clear that the width of the confidence interval increases greatly as the number of cells in dataset decrease. Similar situation is likely to be observed in experimental datasets, where the number of cells obtained from each sample may differ, particularly after quality control. scDC utilizes stratified bootstrap sampling, stratifying based on each subject. Without stratified bootstrap sampling, we will over-estimate the SE - this is a natural consequence of large subject to subject variability. It is not appropriate to assume that all subjects share the same cell-type proportions and be from the same multinomial distribution. In the stratified bootstrap, we assume that each subject has a different cell-type proportion and draws from a different multinomial distribution.

Finally, we implemented scDC as part of the package *scdney* for use by the scientific community. This package performs stratified bootstrap, builds GLM models and provides visualizations of the bootstrap results.

## Conclusions

In this paper, we present scDC, a novel statistical framework for performing differential composition analysis in scRNA-seq experiments. scDC utilizes bootstrap resampling to estimate the standard errors in cell-type proportions, and enables significance testing from bootstrap GLM and GLMM analysis. We applied scDC to both simulation and synthetic data sets and showed that it can accurately recover true cell-type proportions and estimate the variance in cell-type proportions. By pooling estimates from GLM analyses in each bootstrap resample, we also showed that we can perform significance testing of composition differences across subjects and conditions. scDC is implemented as an R package and can be freely downloaded from our GitHub repository. Our implementation supports direct input from sc-RNA expression matrix.

## Methods

### Simulated data generation

The R package *powsimR* was used to simulate the single-cell RNA-seq data [19]. We selected 10% of genes to be differentially expressed between two cell-types, with fold changes sampled from a multivariate normal distribution. Gene specific distributional parameters are estimated by a dataset from mouse embryonic stem cells [20].

*Simulation with one condition*To validate the bootstrapping estimation, we simulated a population of 5000 cells consisting of 4 cells types. Four different simulations were set up, with three simulations consisting of 4 cell-types with proportion of 10%, 20%, 30% and 40% (Simulated data Sim1, Sim2, Sim3), and one simulation with proportion 5%, 25%, 30% and 40% (Simulated data Sim4). Simulated data Sim1 has 10 replicates, with 500 cells in each replicates. Four replicates were randomly chosen and bootstrapped. Simulated data Sim2 has 50 replicates in total, with 100 cells in each replicate. Five replicates were randomly chosen and bootstrapped. Simulated data Sim3 has 100 replicates in total, with 50 cells in each replicate. 20 replicates were chosen and bootstrapped. Simulated data Sim4 has 50 replicates in total, with 100 cells in each replicate. We chose 20 replicates to be bootstrapped (Table 1).

*Simulation with two condition*To validate our scDC procedure in-silico, we simulated a population of 6000 cells of four cell-types, with two conditions with different cell-type proportions. Each condition is divided into three subsets, which can be considered as the subpopulation of three subjects. Then we randomly sampled 200 cells from each subpopulation to create a simulated dataset with 1200 cells. Simulated data SimP1 and SimP2 represents the situation when the biological replicates are available. Here we sample from the same multinomial distribution with true cell-type proportions given in Table 2. Simulated data SimP3 and SimP4 represents the situation where the variability between subjects follows a *N*(0,*σ*^2^) distribution, where *σ*=0.02. The detailed cell-type composition settings of each simulation are listed in Table 2.

*Simulation with rare cell-types and correlated cell-types*We simulated four datasets that have different degree of correlation between cell-types and four datasets that have rare cell-types of different proportions. For each dataset, we first simulated a population of 2400 cells, then we randomly sampled 1200 cells to create the final dataset. Each dataset is made up of two conditions with different cell-type proportions and three replicates in each condition. Variability between subjects are introduced by modelling a *N*(0,*σ*^2^) distribution, where *σ*=0.02. The detailed cell-type composition settings of each simulation are listed in Additional file 1: Table S1.

*Simulation of complex dataset*To test scDC on a dataset with complexity similar to the characteristics of experimental dataset, we simulated one dataset with 8000 cells and 10 cell-types and randomly selected 4000 cells to create the final dataset. The 10 cell-types contains 3 rare cell-types with proportion as low as 1%. This dataset is composed of 2 conditions and 4 replicate subjects in each condition. Variability between subjects are introduced by modelling a *N*(0,*σ*^2^) distribution, where *σ*=0.02. Detailed cell-type composition settings for this simulation are listed in Additional file 1: Table S2.

### Synthetic data

We generated two synthetic datasets from two publicly available datasets [1, 3]. All data were first normalized by TPM (transcripts per million) and log-transformed. Any samples with a total of less than 50 cells of interest or with less than 10 cells of any cell-types of interest were excluded.

*Synthetic Pancreas Dataset*The Pancreas dataset contains 2,209 single cells taken from the pancreas of six healthy and four type 2 diabetic human cadaveric donors (E-MTAB-5061) [1]. Alpha, beta and ductal cells were selected to create the synthetic dataset. Cell labels were provided for all cells by the authors.

*Synthetic Neuronal Dataset*The Neuronal dataset contains 21,465 single cells taken from neural tubes of mouse embryo at five developmental time points from e9.5 to e13.5 [3]. Raw data is made available by the author on their GitHub repository https://github.com/juliendelile/MouseSpinalCordAtlas. The cell-types mesoderm, neural crest, neural crest neurons and progenitor were selected to create the synthetic dataset. Cell-type labels were provided by the original authors through assessing the expression of a list of curated genes.

### Bootstrap confidence intervals

Let *n*_*ij*_ be the number of cells of cell-type *i* and subject *j*, *i*=1,…,*C*,*j*=1,…,*R*; *N*_*j*_ be the number of cells within the subject *j*; and *N* be the total number of cells in the dataset. For a typical subject *j*, the *i*th cell-type proportion is defined as *p*_*ij*_ and calculated by

$$p_{ij} = \frac{n_{ij}}{\sum_{i} n_{ij}} = \frac{n_{ij}}{N_{j}}.$$

*The percentile method*This is an intuitive method for estimating the bootstrap confidence interval by first estimating the empirical distribution of *p*_*ij*_. Let $\hat {p}_{ij(\alpha)}^{*}$ represent the 100*α*-th percentile of *B* bootstrap replications, $\hat {p}_{ij(1)}^{*}$, $\hat {p}_{ij(2)}^{*},\ldots,\hat {p}_{ij(B)}^{*}$, with

$$\hat{p}_{ij(1)}^{*} \leq \hat{p}_{ij(2)}^{*}\leq \hat{p}_{ij(3)}^{*} \leq... \leq \hat{p}_{ij(B)}^{*},$$

where *B*=10000 by default. Then the percentile interval of 100(1−*α*)*%* for *p*_*ij*_ is estimated by

$$\left[\hat{p}_{ij(B\alpha/2)}^{*}, \hat{p}_{ij(B(1-\alpha/2))}^{*}\right].$$

*The BCa method*To account for skewness in the confidence interval, we used the Bias-corrected and accelerated (BCa) confidence intervals proposed by Efron (1987) [21]. The BCa method uses percentiles of bootstrap distribution described above, but depends on an acceleration parameter $\hat {a}$ and a bias-correction factor $\hat {z}_{0}$. The 100(1−*α*)*%* BCa interval of $\hat {p}^{*}_{ij}$ can be calculated by

$$\left[\hat{p}_{ij(\alpha_{1})}^{*}, \hat{p}_{ij(\alpha_{2})}^{*}\right],$$

where

$$\alpha_{1} = \Phi\left(\hat{z}_{0} + \frac{\hat{z}_{0} + z_{(\alpha)}}{1-\hat{a}(\hat{z}_{0} + z_{(\alpha)})} \right);$$

$$\alpha_{2} = \Phi\left(\hat{z}_{0} + \frac{\hat{z}_{0} + z_{(1-\alpha)}}{1-\hat{a}(\hat{z}_{0} + z_{(1-\alpha)})} \right).$$

Here *Φ*(·) is the cumulative distribution function of standard normal distribution, and *z*_(*α*)_ indicates the 100*α*-th percentile point of a standard normal distribution.

This method estimates two adjustment factors. First, the bias-correction factor which estimates the proportion of the bootstrap replication less than the original estimate. This is calculated by

$$\hat{z}_{0} = \Phi^{-1}(\#\{\hat{p}_{ij(b)}^{*}<\hat{p}_{ij}\}/B),$$

where *Φ*^−1^ is the inverse cumulative distribution function of standard normal and $\hat {p}_{ij}$ is the original estimate of proportion, derived from Step 2 of clustering in the scDC procedure.

The second is the acceleration factor which quantifies the rate of change of the standard error of $\hat {p}_{ij}$ with respect to the original estimate *p*_*ij*_. This is calculated using a jackknife approach as follows,

$$\hat{a} = \frac{\sum_{n=1}^{N} (\hat{p}_{ij(\cdot)}-\hat{p}_{ij(n)})^{3}}{6\left\{\sum_{n=1}^{N} (\hat{p}_{ij(\cdot)}-\hat{p}_{ij(n)})^{2}\right\}^{3/2}},$$

where $\hat {p}_{ij(n)}$ is the estimate of *p*_*ij*_ holding out cell *n* in the data, and $\hat {p}_{ij(\cdot)} = \frac {1}{N}\sum _{n=1}^{N} \hat {p}_{ij(n)}$. We modifies the implementation of function *bcanon* in *bootstrap* package to estimate BCa intervals for cell-type proportions of each subject [22].

Note that, for data with only one subject, we can still estimate the CIs with *j*=1.

### Single cell differential composition analysis (scDC)

scDC is a 4-step procedure that tests for differences in cell-type composition between conditions.

*Step 1: Stratified bootstrap*This is used to generate *B* bootstrap samples by sampling with replacement and stratified by subjects. We denote each bootstrap sample data of subject *j* as $Y^{*}_{bj}$. All genes with zero variance in each of the *B* bootstrap sample $Y^{*}_{bj}$ are removed as they are not informative for cell-type determination.

*Step 2: Cell-type identification*We estimate the cell-type identity of each cell by clustering each bootstrap resample $Y^{*}_{bj}$ and estimate the bootstrap cell-type proportions $\hat {p}^{*}_{b1}, \hat {p}^{*}_{b2}, \ldots, \hat {p}^{*}_{bC}$ for all *C* cell-types via clustering The clustering first performed dimension reduction using PCA (R package *stats* [23]) to the first 10 principal component, follow by k-means clustering with Pearson correlation as the similarity measure using function *scClust* in R package *scdney* [7]. The number of clusters, *K*, was initially set to be the number of cell-types *C* in the data. Cluster identity was assigned based on the majority cell-types in a cluster.

In cases where the clustering result do not recover all the cell-types, clustering was re-run with the number of clusters *K* increased by one. We repeated the clustering procedure until each cell-type has been assigned to at least one cluster. We then recorded the number of cells in each cell-type labelled cluster that belongs to each subject or replicate *j*.

*Step 3: Pooled estimates from B complete linear modelanalyses.*For each bootstrap resample $Y^{*}_{bj}$, a Generalized Linear Model was fitted to assess the significance associated with each predictor variable. The R package *lme4* was used to fit the model [24]. Two types of models were considered — the fixed effect model (GLM) and the mixed effect model (GLMM) by treating the subject as a random effect. For the fixed effect model (GLM) the following R code


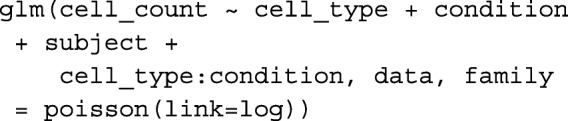
 was used.

For the mixed effect model (GLMM), the following R code


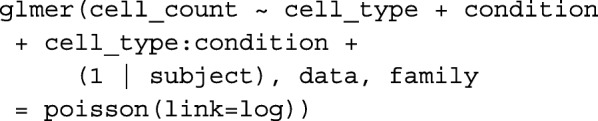
 was used.

This generated *B* bootstrap coefficient estimates $\hat {\boldsymbol {\beta }}^{*}_{1}, \hat {\boldsymbol {\beta }}^{*}_{2}, \ldots \hat {\boldsymbol {\beta }}^{*}_{B}$ from the GLM or GLMM model. *B*=100 by default if user does not require any confidence interval to be estimated, otherwise *B*=10000 by default. The pooled model estimate based on Rubin’s rules [14] is

$$\bar{\boldsymbol{\beta}}^{*} = \frac {1}{B} \sum_{b=1}^{B}{\hat{{\boldsymbol{\beta}}}_{b}^{*}}, $$

where ${\hat {{\boldsymbol {\beta }}}_{b}^{*}} $ is the coefficients estimates from the GLM model fitted with the *b*^*th*^ bootstrap resample. The corresponding estimated pooled standard error is

$${SE}_{pooled}=\sqrt {{V}_{within}+{V}_{between}+\frac {{V}_{between}}{B}},$$

with *V*_*within*_ and *V*_*between*_ denotes the within and between imputation variance respectively. These are calculated by

$${V}_{within} = \frac {1}{B} \sum_{b=1}^{B}{{SE}_{b}^{*2}},$$

with ${SE}_{b}^{*2}$ represents the sum of the squared Standard Error (SE), estimated in each bootstrap dataset *b*. and

$${V}_{between} = \sqrt {\frac {\sum_{b=1}^{B}({{\hat{\boldsymbol{\beta}}^{*}_{b}}-\bar{\boldsymbol{\beta}}^{*})^{2}}}{B-1}}.$$

We used the pool function in the R package *MICE* [25].

*Step 4: Significance testing*For significance testing of any of the pooled parameters of interest such as the interaction effect between cell-types and conditions, the univariate Wald test [14, 25] is used and the test statistics is defined as:

$$W_{pooled} = \frac{\bar{{\beta}}^{*2}_{i}}{SE_{pooled}}, $$

where $\bar {{\beta }}^{*}_{i}$ is the *i*^*th*^ element of the pooled coefficient vector $\bar {\boldsymbol {\beta }}^{*}$.

## Supplementary information

**Additional file 1** Supplementary Tables and Figures. **Table S1.** Simulation cases with correlated and rare cell-types. **Table S2.** Simulation of complex dataset. **Table S3.** Summary from Pooled GLM on neuronal dataset. **Figure S1.** scDC on correlated and rare cell-types. **Figure S2.** scDC on complex dataset. **Figure S3.** Runtime of scDC with varying number of cells and cell-types.

## Data Availability

The method scDC is implemented in R package *scdney*, available from https://github.com/SydneyBioX/scdney. Data used in this paper are available through the following link. The human pancreas dataset is available at http://sandberg.cmb.ki.se/pancreas/. Date accessed 4 September 2019. The mouse neuronal dataset can be found at http://www.ebi.ac.uk/arrayexpress/experiments/E-MTAB-7320. Date accessed 4 September 2019.

## Abbreviations

BCa: Bias-corrected and accelerated
CI: Confidence interval
GLM: Generalized linear model
GLMM: Generalized linear mixed model
scDC: Single cell differential composition
scRNA-seq: Single cell RNA sequencing
SE: Standard error
T2D: Type two diabetes
