# Supplementary material for: scDC: single cell differential composition analysis
Source: BMC Bioinformatics. 2019 Dec 24;20(Suppl 19):721. doi: 10.1186/s12859-019-3211-9 (PMC6929335; doi:10.1186/s12859-019-3211-9)
Supplement: Supplementary file 1 — Additional file 1 Supplementary Tables and Figures. Table S1. Simulation cases with correlated and rare cell-types. Table S2. Simulation of complex dataset. Table S3. Summary from Pooled GLM on neuronal dataset. Figure S1. scDC on correlated and rare cell-types. Figure S2. scDC on complex dataset. Figure S3. Runtime of scDC with varying number of cells and cell-types. [file 12859_2019_3211_MOESM1_ESM.pdf]

Supplementary Material for scDC:Single cell differential composition analysis

Table S1 Simulation cases with correlated and rare cell-types

	Condition 1			Condition 2		
Datasets	p_{11}	p_{21}	sd	p_{12}	p_{22}	sd
Sim S1	0.25	0.75	0.9	0.4	0.6	0.9
Sim S2	0.25	0.75	0.8	0.4	0.6	0.8
Sim S3	0.25	0.75	0.7	0.4	0.6	0.7
Sim S4	0.25	0.75	0.6	0.4	0.6	0.6

	Condition 1			Condition 2		
Datasets	p_{11}	p_{21}	p_{31}	p_{12}	p_{22}	p_{32}
Sim S5	0.7	0.26	0.04	0.6	0.36	0.04
Sim S6	0.7	0.27	0.03	0.6	0.37	0.03
Sim S7	0.7	0.28	0.02	0.6	0.38	0.02
Sim S8	0.7	0.29	0.01	0.6	0.39	0.01

Eight simulation datasets are made, each contains 1200 cells. p_{ij} corresponds to the proportion of cell-type in cell-type i and condition j. Datasets Sim S1 to S4 contains two cell-types with varying degree of correlation. The amount of correlation between two cell-types is controlled by the amount log2 phenotypic fold changes for differentially expressed genes. In the simulation datasets, the fold change for each gene is set by drawing a random number with mean 0 and a particular sd specified in this table. Datasets Sim S5 to S8 contains three cell-types, with cell-type 3 being rare cell-type.

Table S2 Simulation of complex dataset

	Condition 1									
Dataset	p_{11}	p_{21}	p_{31}	p_{41}	p_{51}	p_{61}	p_{71}	p_{81}	p_{91}	p_{10_1}
Dataset 1	0.03	0.03	0.03	0.07	0.11	0.11	0.13	0.17	0.16	0.16

	Condition 2									
Dataset	p_{12}	p_{22}	p_{32}	p_{42}	p_{52}	p_{62}	p_{72}	p_{82}	p_{92}	p_{10_2}
Dataset 1	0.03	0.04	0.05	0.08	0.11	0.12	0.14	0.18	0.13	0.12

One simulation dataset containing 4000 cells was generated. p_{ij} corresponds to the proportion in cell-type i and condition j. This dataset is composed of 10 cell-types with the first three cell-types being rare populations. Using the proportion listed in the table, we simulated four subjects per condition. Since subject variability was introduced, some of these proportions were as low as 1% for some subjects.

Table S3 Summary from Pooled GLM on neuronal dataset

	Fixed effect model - Neuronal				
	estimate	std.error	statistic	df	p.value
(Intercept)	7.22	0.03	258.38	14.47	0.00
Neural crest	-1.92	0.08	-25.23	8.41	0.00
Neural crest neurons	-3.13	0.18	-17.64	4.78	0.00
Progenitor	-1.08	0.06	-17.22	6.22	0.00
e10.5	-0.56	0.05	-11.90	12.93	0.00
e11.5	-2.07	0.08	-26.27	11.06	0.00
e12.5	-2.68	0.08	-32.06	12.45	0.00
e13.5	-2.88	0.09	-31.32	12.62	0.00
m_e9.5_2	0.18	0.03	6.11	19.25	0.00
m_e10.5_1	0.16	0.03	4.76	19.25	0.00
m_e11.5_1	0.34	0.04	7.79	19.25	0.00
m_e12.5_1	0.45	0.05	9.03	19.25	0.00
m_e12.5_2	0.13	0.05	2.38	19.25	0.03
m_e13.5_1	0.47	0.06	7.96	19.25	0.00
m_e13.5_2	0.14	0.06	2.26	19.25	0.04
Neural crest:e10.5	0.16	0.14	1.15	6.15	0.26
Neural crest neurons:e10.5	1.45	0.22	6.64	4.66	0.00
Progenitor:e10.5	0.72	0.09	8.36	6.86	0.00
Neural crest:e11.5	1.72	0.13	13.05	8.62	0.00
Neural crest neurons:e11.5	2.78	0.30	9.39	3.01	0.00
Progenitor:e11.5	2.11	0.12	17.31	5.78	0.00
Neural crest:e12.5	2.15	0.15	14.54	6.64	0.00
Neural crest neurons:e12.5	3.56	0.21	17.21	5.60	0.00
Progenitor:e12.5	2.23	0.12	19.24	7.12	0.00
Neural crest:e13.5	2.11	0.14	15.12	8.59	0.00
Neural crest neurons:e13.5	2.95	0.22	13.60	6.25	0.00
Progenitor:e13.5	2.21	0.11	19.34	8.51	0.00

	Mixed effect model - Neuronal				
	estimate	std.error	statistic	df	p.value
(Intercept)	7.31	0.11	64.82	24.82	0.00
Neural crest	-1.92	0.08	-25.23	10.93	0.00
Neural crest neurons	-3.13	0.18	-17.64	6.20	0.00
Progenitor	-1.08	0.06	-17.22	8.07	0.00
e10.5	-0.57	0.16	-3.54	24.51	0.00
e11.5	-1.99	0.17	-11.56	22.97	0.00
e12.5	-2.58	0.16	-15.98	22.83	0.00
e13.5	-2.76	0.16	-16.82	22.50	0.00
Neural crest:e10.5	0.16	0.14	1.15	7.99	0.26
Neural crest neurons:e10.5	1.45	0.22	6.64	6.06	0.00
Progenitor:e10.5	0.72	0.09	8.36	8.90	0.00
Neural crest:e11.5	1.72	0.13	13.06	11.19	0.00
Neural crest neurons:e11.5	2.78	0.30	9.39	3.91	0.00
Progenitor:e11.5	2.11	0.12	17.31	7.50	0.00
Neural crest:e12.5	2.15	0.15	14.54	8.61	0.00
Neural crest neurons:e12.5	3.56	0.21	17.21	7.26	0.00
Progenitor:e12.5	2.23	0.12	19.24	9.24	0.00
Neural crest:e13.5	2.11	0.14	15.13	11.16	0.00
Neural crest neurons:e13.5	2.95	0.22	13.60	8.11	0.00
Progenitor:e13.5	2.21	0.11	19.35	11.04	0.00

Neural crest, neural crest neurons and progenitor are the cell-type effect. e10.5, e11.5, e12.5, e13.5 are the developmental time points. These are the condition effect. m_e9.5_2 indicate subject effect, where m stands for mouse sample, 2 stands for the subject ID in time point e9.5.

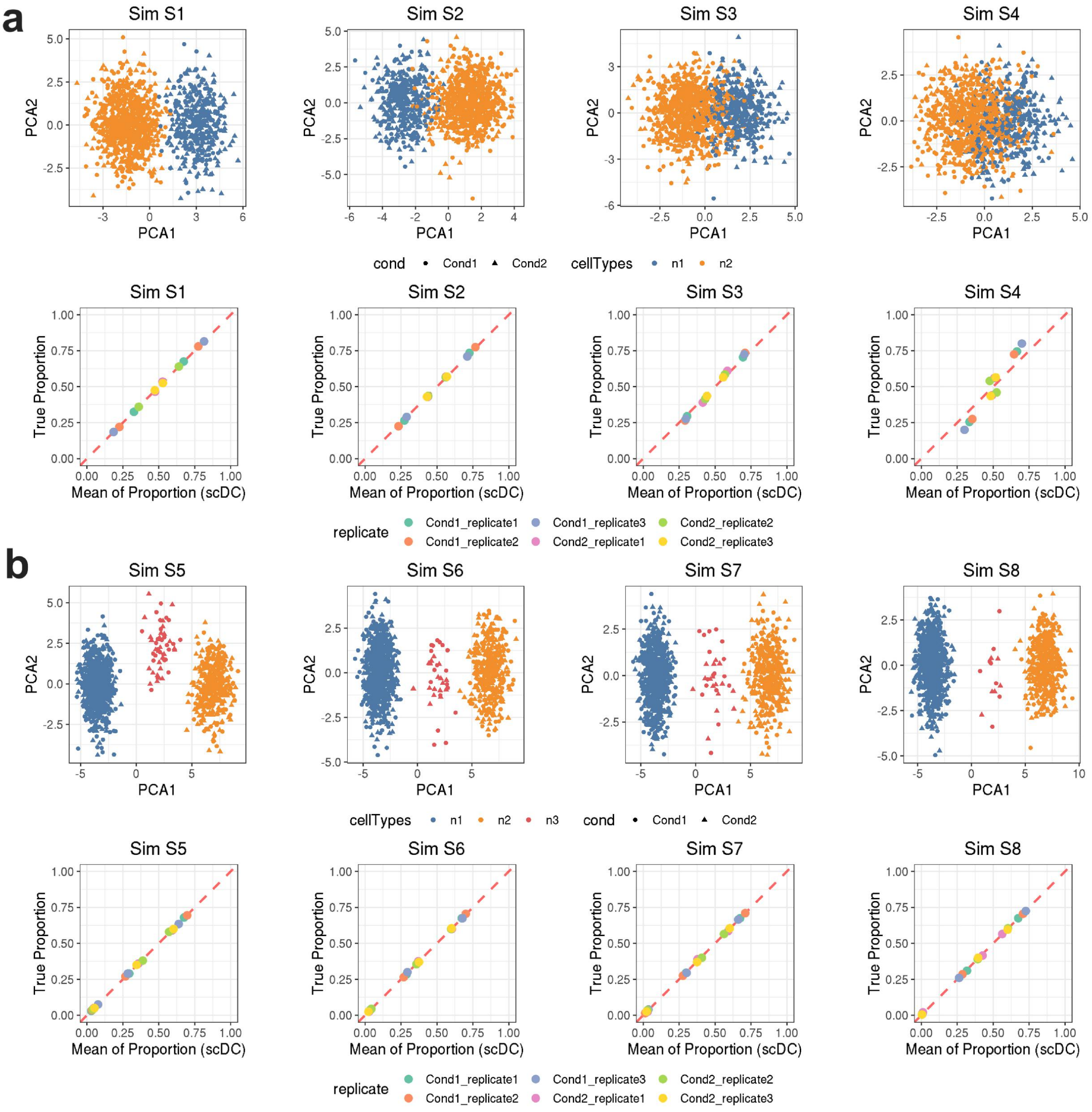

Figure S1. scDC on correlated and rare cell-types scDC has been tested on performance on simulated datasets with correlated cell-types and rare cell-types. a shows result on four datasets S1 - S4 containing correlated cell-types. Top row shows t-SNE visualisation of each dataset and bottom row shows scatter plot of mean proportion estimate calculated from scDC (x-axis) and true proportion from the simulation data (y-axis). The result demonstrates that scDC is still highly accurate when the extent of overlap is moderate. **a** large amount of overlap does not severely impact the performance of scDC either. **b** shows result on four datasets S5 - S8 containing rare cell-types. Top row shows t-SNE visualisation of each dataset and bottom row shows scatter plot of mean proportion estimate calculated from scDC (x-axis) and true proportion from the simulation data (y-axis). Across all four datasets, all dots lie on the diagonal line, even when the proportion of a cell-type is 1%. This reveals that scDC is highly accurate in estimating rare cell-types

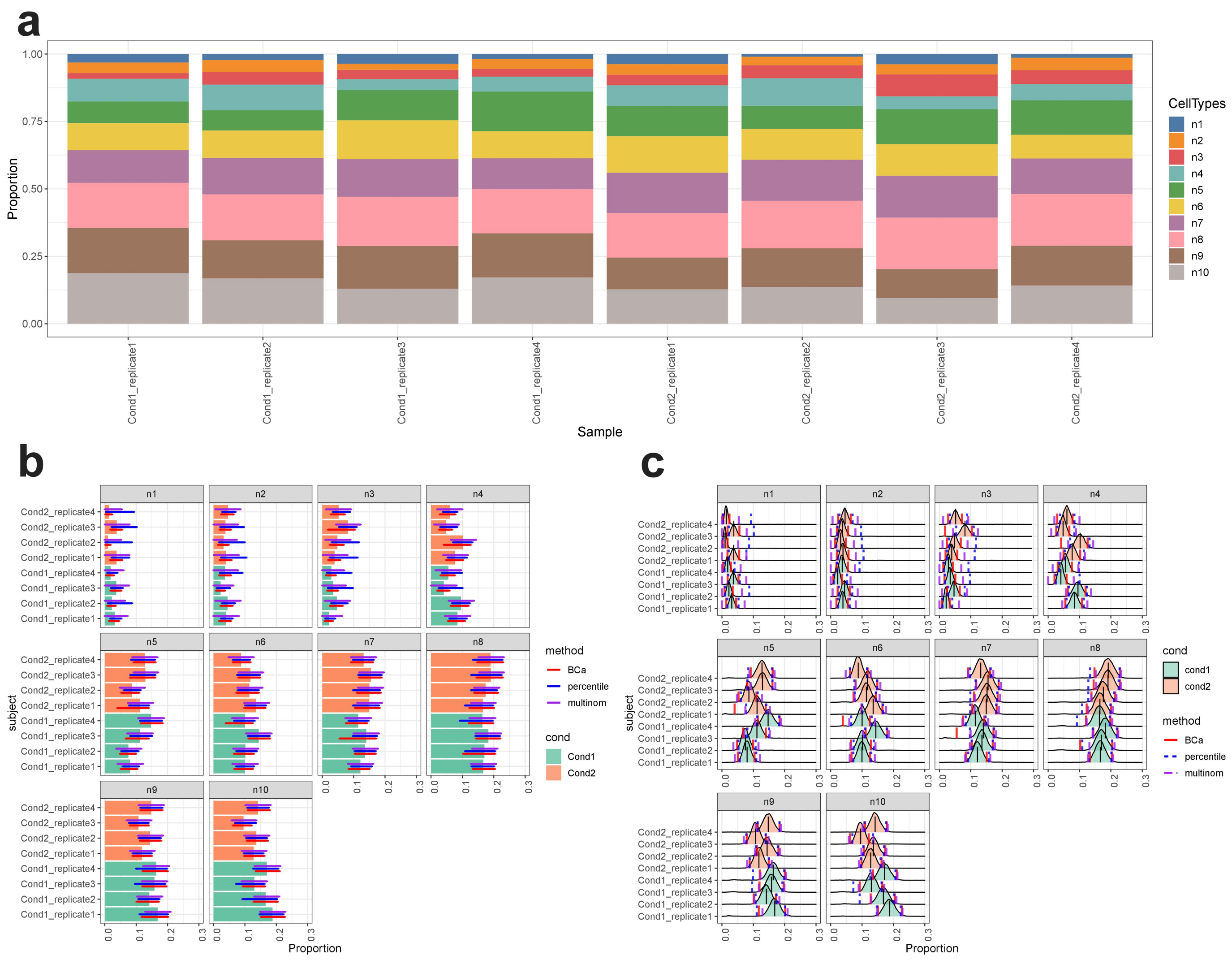

Figure S2. scDC on complex dataset We tested the performance of scDC on a complex simulated dataset containing 4000 cells, 10 cell-types, two conditions and four samples in each condition. **a** shows the true cell-types proportion for each of the eight samples in this dataset. **b** demonstrates the proportion estimates produced from scDC and the associated confidence intervals. **c** shows the density distribution of proportion estimates from 10,000 bootstraps. The vertical line indicates the true cell-types proportion.

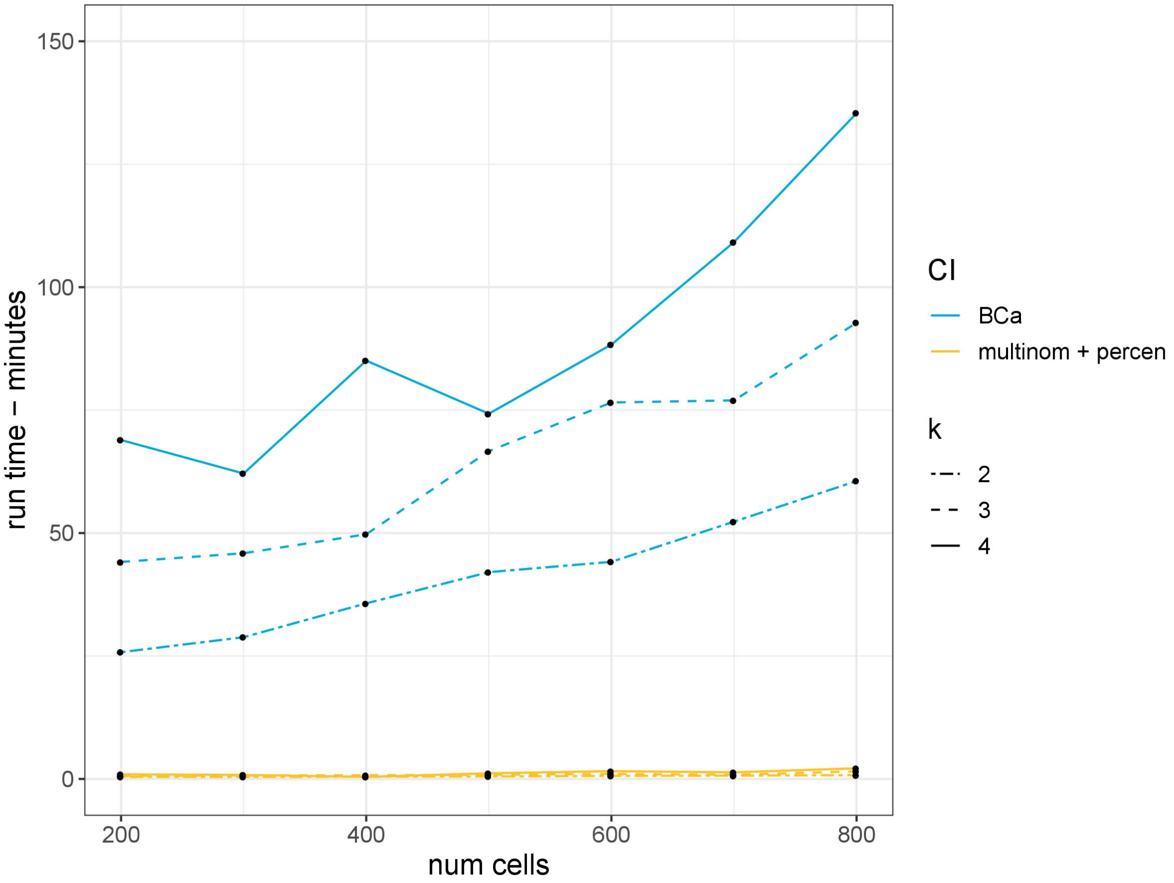

Figure S3. Runtime of scDC with varying number of cells and cell-types We evaluated scDC implementation using a CPU system with Debian version 9.9. The machine has 256GB main memory and 2 of Intel Xeon E5-2690v4 2.60 GHz processor, with 14 cores in each. We used 10 cores for evaluation. Three simulation datasets containing 2, 3 and 4 cell-types were generated for evaluation. We also tested the impact of size of dataset by randomly selecting 200 to 800 cells to evaluate. The runtime clearly shows that both percentile and multinomial CI could be completed in less than 2 minutes for all datasets tested. Whilst BCa is slower, the runtime nevertheless increases only linearly with increasing number of cell-types and cells.
